# Quality of sleep and burnout among undergraduate medical students at the university of Nairobi, Kenya

**DOI:** 10.1192/bjo.2021.742

**Published:** 2021-06-18

**Authors:** Linda Nyamute, Muthoni Mathai, Anne Mbwayo

**Affiliations:** 1 University of Nairobi; 2 Consultant Psychiatrist and Senior lecturer, University of Nairobi; 3 Clinical psychologist and lecturer, University of Nairobi

## Abstract

**Aims:**

The main objective was to determine whether quality of sleep is associated with burnout among undergraduate medical students at the University of Nairobi.

The null hypothesis in our study population was; 'There is no significant association between poor sleep quality and burnout'.

**Background:**

In a pressure prevailing environment, medical students find themselves in a vicious cycle of cutting down on sleep in attempts to cope and adjust to increasing workloads. Students with poor sleep quality have been found to perform worse in their board exam and have strained social engagements. Ultimately, this chronic sleep deprivation may lead to burnout which may cause diminished sense of accomplishment and impaired professional conduct, that may be carried on to the career as a physician. High levels of burnout have been associated with suicides.

**Method:**

The sample size obtained was 384 and participants were selected by a mixed sampling method. Data collection was through self-administered questionnaires. Scales used for this study were the Pittsburg Sleep Quality Index(PSQI) and the Oldenburg Burnout Inventory(OLBI).

Ethical considerations were adhered to and approval obtained from the Kenyatta National Hospital-University of Nairobi(KNH-UON) Ethics Board. Data entry and analysis was by SPSS v23. Data from 336 questionnaires were deemed fit for analysis.

**Result:**

With a response rate of 87.5%, the prevalence of poor sleep quality and burnout were 69.9% and 74.7% respectively. There was a significant positive association between poor sleep quality and female gender, clinical years of study, living with family, poorly perceived socio-economic state and poor subjective academic performance. In addition, being female, younger, pre-clinical years, living independently off-campus and poor subjective academic performance were significantly associated with higher levels of burnout.

Burnout had a significant correlation with poor sleep quality. Daytime functioning, a component of sleep quality had the highest correlation with components of burnout, disengagement and exhaustion. Overall, 57% of the respondents had both poor sleep quality &burnout, while only 12% were good sleepers with no burnout. Furthermore, having poor sleep increased the risk of having burnout by 2.8times. It is crucial that students adopt better sleeping habits to reduce the risk of burnout.

**Conclusion:**

With the high prevalence of poor sleep quality and burnout, peer-support groups and peer-led mentorship programs are recommended within this population to help deal with expectations, challenges and difficulties encountered within the course of medical education, in addition to preparing for the early future careers.

